# Macrophage Plasticity in Skeletal Muscle Repair

**DOI:** 10.1155/2014/560629

**Published:** 2014-04-17

**Authors:** Elena Rigamonti, Paola Zordan, Clara Sciorati, Patrizia Rovere-Querini, Silvia Brunelli

**Affiliations:** ^1^Division of Regenerative Medicine, Stem Cells and Gene Therapy, San Raffaele Scientific Institute, Via Olgettina 58, 20132 Milano, Italy; ^2^San Raffaele University, Via Olgettina 58, 20132 Milano, Italy; ^3^Department of Health Sciences, University of Milano-Bicocca, via Cadore 48, 20900 Monza, Italy

## Abstract

Macrophages are one of the first barriers of host defence against pathogens. Beyond their role in innate immunity, macrophages play increasingly defined roles in orchestrating the healing of various injured tissues. Perturbations of macrophage function and/or activation may result in impaired regeneration and fibrosis deposition as described in several chronic pathological diseases. Heterogeneity and plasticity have been demonstrated to be hallmarks of macrophages. In response to environmental cues they display a proinflammatory (M1) or an alternative anti-inflammatory (M2) phenotype. A lot of evidence demonstrated that after acute injury M1 macrophages infiltrate early to promote the clearance of necrotic debris, whereas M2 macrophages appear later to sustain tissue healing. Whether the sequential presence of two different macrophage populations results from a dynamic shift in macrophage polarization or from the recruitment of new circulating monocytes is a subject of ongoing debate. In this paper, we discuss the current available information about the role that different phenotypes of macrophages plays after injury and during the remodelling phase in different tissue types, with particular attention to the skeletal muscle.

## 1. Role of Macrophages in Inflammation Resolution and Tissue Remodelling

Macrophages are essential for the efficient healing of numerous tissues. They contribute to homeostatic tissue remodelling during foetal life [1, 2] and in several tissues in the adult. The healing process consists of overlapping phases of inflammation, tissue formation, and remodelling with reorganization of vasculature and extracellular matrix. Macrophages participate in all the different phases of tissue repair: they can promote phagocytosis of cellular debris and apoptotic neutrophils and produce cytokines that may help orchestrate the healing response. However, due to the release of proinflammatory cytokines and cytotoxic radical species, uncontrolled activity of macrophages may also be detrimental to tissue repair. Indeed, several human diseases are characterized by attenuated repair responses and imbalances in the inflammatory response with increased number of infiltrating macrophages [3–5]. Heterogeneity and plasticity of macrophages could explain these apparently contrasting roles in tissue healing. All macrophages express common markers such as CD11b (Mac1 or CR3), CD68, and CD115 (M-CSF receptor). However, at least two distinct macrophage populations have been identified: the classically activated M1 phenotype and the alternative activated M2 phenotype [6]. Classically activated M1 macrophages are induced* in vitro* by IFN*γ*, alone or in concert with microbial stimuli (e.g., LPS) or selected cytokines (e.g., TNF and GM-CSF). They have proinflammatory functions: they produce effector molecules (reactive oxygen and nitrogen intermediates) and inflammatory cytokines (IL-1*β*, TNF*α*, and IL-6) and participate as inducer and effector cells in polarized Th1 responses. Alternatively activated M2 macrophages comprise cells exposed to low concentrations of M-CSF in the presence of IL-4, IL-13, or IL-10. They participate in polarized Th2 reactions, parasite clearance, damping of inflammation, and promotion of angiogenesis and tissue remodelling [7, 8].* In vivo*, the identification of macrophage phenotype is complicated since macrophages are obviously exposed to a microenvironment that is more complex respect to cell culture conditions, and they display characteristics that do not conform to the* in vitro* defined phenotypic categories. Therefore, the* in vivo* classification of macrophages in two polarized states (M1 versus M2) sounds as an oversimplification. Therefore, in the last years characterization of macrophage phenotype* in vivo* during tissue repair has been a matter of active investigation. Macrophage activation has been described as a dynamic process: the same cell may initially induce proinflammatory and cytotoxic reactions and later may take part in the resolution of inflammation and wound healing [9]. A common scenario is emerging, in which soon after injury infiltrating macrophages are mainly proinflammatory M1 macrophages, whereas M2 macrophages are the primary effectors of later stages of tissue repair or remodelling phases [10–12]. Recent evidence has also shed light on the functional cross-talk between macrophages and stem/progenitor cells, which may contribute to repair and remodelling in different tissue/organs [13–15].

Specific examples of the origin and functions of macrophages during healing of various tissues are illustrated below, with particular emphasis on skeletal muscle.

Macrophages play a crucial role in the restoration of skin integrity and homeostasis and exert distinct functions during the multiple phases of skin repair, despite the underlying molecular mechanisms remaining partially unclear [16, 17]. Transgenic mice that express the human diphtheria toxin receptor (DTR) under the control of the CD11b promoter have been shown to allow a conditional depletion of macrophages [18]. Using these transgenic mice, Mirza et al. demonstrated that macrophage depletion during wound healing leads to delayed reepithelialization, reduced collagen deposition, impaired angiogenesis, and finally wound closure [17]. Interestingly, Lucas et al. showed that during the early phases of skin repair, infiltrating macrophages are alternatively activated and express high levels of growth factors, VEGF*α* and TGF*β*, which contribute, respectively, to wound angiogenesis and myofibroblast differentiation [16]. During the midstage of the skin repair response, macrophages still express VEGF*α* and TGF*β* but to a lesser extent and they are crucial for vessels stabilization and scar formation. More recently, TGF*β* has been described to regulate wound healing through TLR4 receptor. Indeed, TLR4^−/−^ mice display impaired skin wound healing with decreased macrophage infiltration and reduced levels of TGF*β* [19].

Dynamic changes in monocyte/macrophage phenotype have been described also in a model of myocardial injury. Macrophages have been suggested to be beneficial for myocardial wound healing. Optimum outcome of myocardial injury is strictly related to the balance between debris clearance and myocardial extracellular matrix repair. Liposome-mediated depletion of infiltrating macrophages after myocardial injury results in persistence of cellular debris, impaired vascularization, and myofibroblast infiltration and ultimately leads to ineffective scar formation. After injury, macrophage-depleted mice display cardiac complications and ultimately a significant decreased survival [20]. Two different kinds of monocyte/macrophages populations have been suggested to infiltrate the heart after injury: Ly-6C^high^ proinflammatory monocytes firstly arrive via CCR2 receptor and scavenge necrotic debris; subsequently Ly-6C^low^ preferentially accumulate and promote an anti-inflammatory response and granulation tissue formation [21]. Similar kinetics of monocyte infiltration has been observed also in patients with acute myocardial infarction [22]. Moreover, microarray analyses on RNA of macrophages isolated from infarcted tissues confirmed the expression of proinflammatory (M1) markers in the tissue early after injury and of alternative activated (M2) macrophage markers later during scar tissue formation [23].

The plasticity of macrophages has been reported to play a role also in parenchymal organ diseases, such as liver or lung fibrosis. Liver fibrosis is a common consequence of chronic liver disease and current evidence suggests that this process is mainly driven by a local inflammatory response [24, 25]. Experimental models of liver fibrosis highlight the importance of hepatic resident macrophages, the Kupffer cells, for sustaining inflammation as well as activating the hepatic stem cells (HSC) [26]. However, fibrosis largely depends on recruitment of monocytes into the liver [18, 27]. In a reversible model of liver fibrosis two functionally distinct types of macrophages have been demonstrated to regulate the outcome of the fibrotic response [18]: during the injury phase, infiltrating macrophages promote myofibroblast proliferation and matrix deposition by secreting high amounts of TGF*β* and TNF*α*, whereas during the recovery phase they sustain matrix degradation, probably by releasing MMP13 [28]. Profibrogenic macrophages have been shown to derive mainly from circulating Ly-6C^high^ proinflammatory monocytes, which massively invade injured liver via the CCR2 receptor both in mice and in humans [29–31]. Similarly, a critical role of macrophages in regulating lung fibrosis has been recently described. Evidence supports the involvement of alternative activated macrophages (M2) in lung fibrotic response via secretion of TGF*β* [32]. These results were corroborated by recent observational studies in humans which highlight the presence of M2 macrophages markers in lung diseases: CD163, CCL18, CCL22, and CD206 [33, 34].

## 2. A Case for Macrophages in Acute and Chronic Muscle Damages

The plasticity of macrophages in response to environmental cues has been largely investigated in the skeletal muscle [35–37]. Muscle inflammation is a common physiologic response to exercise and a typical feature of acute and chronic muscle damages. Muscle regeneration and healing after damage mainly depend upon quiescent muscle stem cells, the satellite cells, localized between the basal lamina and the muscle fiber membrane [38]. Upon muscle injury, satellite cells activate, start proliferating, and, subsequently, differentiating into new myotubes that replace damaged muscle [39, 40]. Beside satellite cells, the inflammatory cells that infiltrate the injured muscle deeply influence the outcome of muscle regeneration.

### 2.1. Acute Muscle Injury and Macrophage Activation

Skeletal muscle sterile injury triggers a potent inflammatory response characterized by a rapid and sequential invasion of leukocyte populations that persist during muscle repair, regeneration, and growth. The regeneration process includes an initial proinflammatory phase characterized by release of cytokines and chemokines which promote infiltration of immune cells to the site of damage in order to remove cellular debris [41].

Neutrophils are the first leukocyte population in damaged tissue. They appear within 2 h of muscle damage, reaching a maximum in concentration between 6 and 24 h postinjury and then rapidly decreasing. The actual role of neutrophils in damaged skeletal muscle is still debated. They release molecules (proteolytic enzymes, oxygen-derived reactive species) that may contribute to muscle membrane lysis and, therefore, to damage extension [42]. However, neutrophils have also been suggested to facilitate muscle regeneration by removing tissue debris from the injured area as well as by activating satellite cells [43]. Recent results indicated that the supportive and/or deleterious effects of neutrophils on skeletal muscle might rely on the degree of their activation. Indeed, during modified mechanical loading, neutrophils are efficiently eliminated with no significant muscle fiber injury. Conversely, the presence of microbial products leads to significant neutrophil infiltration and muscle fiber damage [44].

Shortly after neutrophil invasion, macrophages begin to accumulate and, subsequently, become the dominant leukocyte population [45, 46]. They are mainly derived from blood monocytes that have crossed the vessel endothelial barrier to reach the tissue [47]. Macrophages are professional scavengers of apoptotic cells and debris and produce a pattern of signals involved in myogenic precursors activation, matrix remodelling, and neovessel formation [48, 49].* In vivo* studies have unequivocally shown that macrophages play a pivotal role in the muscle repair process [15, 50–54]. Indeed, data from several models of muscle injury (hindlimb ischemia, freeze-injury, unloading/reloading sequences, and myotoxic agent injections) indicate that impairment of macrophage recruitment in injured muscle results in delayed tissue regeneration in terms of appearance of regenerating centronucleated myofibers and persistence of intramuscular adipocytes and fibrosis [55]. More recently, other cell types, including eosinophils and fibroadipogenic precursors, have been shown to contribute to the rapid clearance of necrotic debris and, subsequently, proper muscle regeneration [56].

During the early stages of acute muscle injury, infiltrating and muscle-resident macrophages associated with the epimisyal and perimysial connective tissue contribute in locally attracting monocytes from the blood by secreting chemokines, such as MCP1/CCL2 [57]. Indeed, the expression of MCP1/CCL2 receptor (CCR2) on bone marrow derived cells is critical for normal skeletal muscle regeneration. Mice defective for CCR2 (CCR2^−/−^) display severe impairments in macrophage recruitment and skeletal muscle regeneration following cardiotoxin (CTX)-induced injury [58]. Interestingly, MCP1^−/−^ mice exhibit an intermediate phenotype compared with CCR2^−/−^ mice in terms of macrophage recruitment to the site of injury, resolution of necrosis, and muscle regeneration, thus suggesting that other chemokines, in addition to MCP1, may activate CCR2-dependent regenerative processes [59]. Similarly, CXCL16 has also been shown to regulate monocyte/macrophage entry into the injured muscle [60]. Genetic disruption of CXCL16 pathway resulted in defective homing of macrophages and persistent infiltration of neutrophils, leading to sustained inflammation, impaired muscle regeneration, and scar deposition.

Two different macrophage populations have been described in injured/regenerating skeletal muscle. Arnold et al. [61] identified a population of circulating monocytes, which are selectively recruited to the site of damage and display a proinflammatory phenotype. They secrete inflammatory signals, including TNF*α*, IL-1*β*, and MCP1, and dispose of fiber remnants. Moreover, macrophages infiltrating damaged muscle have been recently shown to express inducible nitric oxide synthase (iNOS), a typical marker of M1 macrophages [62]. The phagocytosis of either apoptotic or necrotic myogenic cells apparently sustains the functional polarization of macrophages towards an anti-inflammatory phenotype. M2 macrophages contribute to dampen the inflammatory response by secreting TGF*β* and IL-10. Moreover, they sustain fiber reconstitution by secreting cytokines that may play a trophic function, such as IGF1 and IL-10. In particular, IL-10 is mainly produced by infiltrating macrophages and its secretion is necessary to sustain viability and allow differentiation and fusion of the myogenic progenitor mesoangioblasts into terminally differentiated myofibers [15]. The sequential presence of proinflammatory and then anti-inflammatory macrophages has been also demonstrated in human muscles. Both subsets of macrophages have been identified in injured/regenerating human muscles. Macrophages expressing M1 markers preferentially associate with proliferating satellite cells, whereas at the time of myogenic differentiation macrophages mainly express anti-inflammatory M2 markers [63].

The cellular and molecular pathways involved in the regulation of macrophage phenotype transition during muscle injury/regeneration have been deeply investigated in the latest years. The cAMP response element-binding protein (CREB) has been demonstrated to be a crucial transcription factor for the upregulation of M2-associated gene while repressing M1 activation. Deletion of two CREB binding sites from the C/EBP*β* gene promoter blocks the downstream induction of anti-inflammatory genes associated with M2-like macrophage activation, whereas the inflammatory (M1) genes are not affected. Upon muscle injury, mice carrying the mutated C/EBP*β* promoter efficiently clear injured muscle from necrotic debris but display severe defects in muscle fiber regeneration, thus confirming that the persistence of inflammatory macrophages in damaged muscle of these mice is not sufficient for effective regeneration [64]. Another molecule playing a key role in regulating macrophage phenotypic transition and muscle recovery is the MAP kinase phosphatase (MKP)-1 [65]. Gene-expression analyses on sorted MKP-1^−/−^ muscle macrophages indicated that MKP-1 controls the inflammatory response as well as the switch from early pro- to late anti-inflammatory macrophage phenotype via p38 MAPK downregulation. Mice deficient in MKP-1 display defective muscle regeneration with persistence of damage and impaired growth of regenerating myofibers. Interestingly, this phenotype could be completely restored by MKP-1^+/+^ bone-marrow transplantation, strongly suggesting dispensability of this protein for satellite cell-dependent myofiber repair [65]. Recently, AMP-activated protein kinase (AMPK)-*α*1 has also been demonstrated to play a significant role in the regulation of macrophage skewing during skeletal muscle regeneration. Increase in AMPK activity has been associated with a decreased proinflammatory status of macrophages [66]. Indeed AMPK*α*1^−/−^ macrophages fail to adopt an anti-inflammatory (M2) phenotype and display a defect in the phagocytic activity [67]. Consistently, mice bearing a specific deletion of AMPK*α*1 in myeloid-cells show a significant delay in skeletal muscle regeneration paralleled by a decreased number of M2 macrophages [67]. More recently, a population of regulatory T cell (Treg) has been shown to infiltrate injured muscles and support muscle repair by modulating the several steps of the regeneration process. Interestingly, muscle Treg cells promote the switch between pro- and anti-inflammatory macrophages; the precise mechanisms and the potentially responsible molecules are currently under investigation [68].

### 2.2. Chronic Muscle Injury

The study of the molecular mechanisms underlying the role of macrophage subpopulations in muscle repair after acute muscle injury could blaze new trails in the comprehension of onset and progression of chronic muscle diseases, even if in these conditions macrophages may exert a more complex role, in response to a more complex and heterogeneous scenario.

*In genetic diseases of the muscle*, such as the* muscle dystrophies*, the noxa cannot be eliminated. The genetic defect usually affects the structure of the muscle fiber: membranes become more fragile, leading to necrosis [69]. Since the stem cell compartment undergoes a progressive depletion/exhaustion and necrosis does not abate, the tissue architecture is progressively disrupted [70]. In addition, the release of adjuvant stimuli, that activate the innate and acquired immune responses, and the generation of reactive oxygen and nitrogen species, may impinge on macrophage survival/polarization and function [54, 68, 71].

Several mouse models of chronic muscle damage exist and allow a better understanding of the role of macrophage plasticity during the onset and progression of diseases. Moreover they are essential in developing a new pharmacological or stem cell based clinical strategy.

In the* mdx* mice, a model for Duchenne muscle dystrophy, the early stage of the disease is characterized by an innate immunity response that is similar to that occurring after an acute injury, with a massive invasion of neutrophils and M1-like macrophages. The classical activation of M1 macrophages is driven by proinflammatory Th1 cytokines, especially TNF*α* and IFN*γ*. Both cytokines are highly expressed in* mdx* muscles and they possibly promote muscle damage during the acute stage of the pathology [37, 72]. Antibody and pharmacological blockade of TNF*α* in young* mdx* mice results in a delayed and significantly reduced amount of skeletal muscle damage [73, 74]. IFN*γ* stimulation of macrophages isolated from* mdx* muscles significantly increases muscle cell lysis* in vitro* [72]. However,* in vivo* ablation of IFN*γ* in young* mdx* mice does not affect muscle fiber damage and only partially reduces iNOS expression without decreasing macrophage cytotoxicity [75]. Classically activated M1 macrophages persist in the dystrophic muscle due to the unremitting inflammatory response and induce further muscle damage through the production of cytotoxic levels of nitric oxide (NO) by iNOS [72].

The role of NO in the muscle is nevertheless more complex. The lack of dystrophin [76, 77] disrupts indeed the recruitment of another nitric oxide synthase isoform, the neuronal NOS (nNOS), to the sarcolemma, thus affecting NO production in muscle fibers [78, 79] and contributing to the severity of the dystrophic phenotype [80, 81]. The rescue of function in* mdx* or dystrophin/utrophin double-knockout mice by overexpressing an nNOS transgene has demonstrated that NO controls disease progression and corrects the balance in macrophage subpopulations [82, 83]. In dystrophic mice the early M1 invasion is indeed followed by the recruitment of a subpopulation of M2 macrophages, expressing CD206, IL-10, and Arginase, that are referred to as M2a; these cells reduce NO mediated cytotoxicity of M1 macrophages by competing for the substrate arginine [75, 84]. Subsequent invasion of the dystrophic muscle by another subpopulation of alternatively activated macrophages, defined as M2c and expressing CD163, further contributes to M1 deactivation and is associated with tissue healing and progression to the regenerative phase [72, 84]. The persistence of inflammation at later stages promotes excessive connective tissue deposition that leads to muscle fibrosis, characteristic of dystrophy [84].

In the presence of the nNOS transgene a decrease in M2c macrophages in the muscle of dystrophic mice was observed, paralleled by a significant reduction of fibrosis. The nNOS transgene has no effect on the concentration of cytolytic M1 macrophages [83].

The role of NO in modulating the inflammatory response in the dystrophic muscle has been demonstrated by treating another mouse model of dystrophy, the alpha-SG KO mouse, with the NO donor Molsidomine [85, 86]. Molsidomine administration leads to a reduction of the inflammatory infiltrate, in particular in terms of number of neutrophils and classically activated macrophages. In addition, most of remaining macrophages coexpress both markers of classical and alternative activation (CD206+ CD163+ CD86+) and might represent a transitional population, which maintains the ability to sustain the proliferation and differentiation of myogenic precursors without contributing to the deposition of collagen and persistence of fibrosis [8, 37].

*Inflammatory myopathies* are another class of chronic muscle diseases. They are heterogeneous and classically comprise* polymyositis* [52],* dermatomyositis* (DM), and* sporadic inclusion body myositis* (IBM) [87]. Despite these disorders differing in prognosis and response to treatment, common clinical signs are muscle mononuclear cells infiltration and myofiber degeneration [88]. Important immunological features include also autoantibodies and autoreactive T lymphocytes with the overexpression of major histocompatibility complex class I molecules on the surface of fibers [89]. In DM the humeral immunity due to CD4^+^ cells and B cells plays a predominant role, while PM and IBM disorders are mediated by cytotoxic CD8^+^ T cells which attack skeletal muscle fibers [88, 90]. Interestingly, macrophage infiltration is common in all inflammatory myopathies. At present, few data are available concerning the phenotype and the role of macrophages in the pathology of inflammatory myopathies. Analyses of muscle biopsies demonstrated that in areas of severe inflammation and necrosis, macrophages express both proinflammatory and anti-inflammatory markers. Indeed, in PM, macrophages are highly positive for iNOS and TGF*β*, thus suggesting the existence of two possible macrophage subpopulations, which could modulate the inflammatory response [91]. Moreover, Reimann et al. demonstrated that the macrophage migration inhibitory factor (MIF) is highly expressed in muscle samples of human PM. MIF is a T cell and macrophage derived proinflammatory cytokine with antiapoptotic, proproliferative, and chemotactic effects. In muscle biopsies of PM, MIF has been detected not only in inflammatory cells but also on muscle fiber membrane, thus suggesting a potential role of MIF in the onset of the disease [92]. In addition to the classical PM, DM, and sporadic IBM,* immune-mediated necrotizing myopathy* (IMNM) is another important class of immune-mediated myopathies [93]. More recently, it has been defined as a Th1-M1-mediated disease due to high levels of proinflammatory cytokines IFN*γ*, TNF*α*, and IL-12 that have been detected in biopsy specimens; by contrast no difference was observed for markers of alternative activation of macrophages between patients and healthy control biopsies [94]. Further investigations are required to better characterize the molecular mechanism of the immune response in inflammatory myopathies and ultimately to design potential therapeutic approaches.

## 3. Conclusions

**Figure 1:**
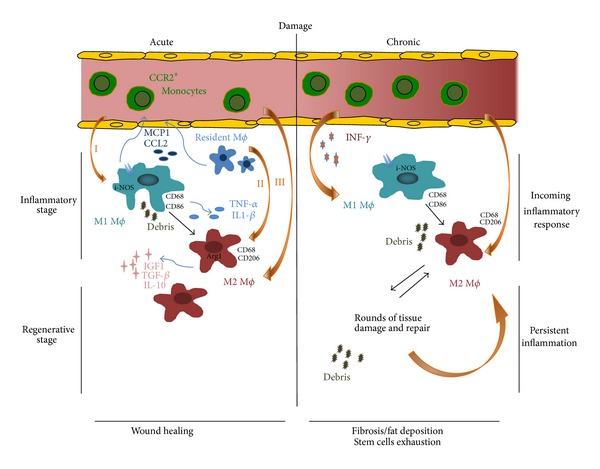
Macrophages in acute and chronic muscle damage. The innate immune system through M1 macrophages activates an inflammatory response: secretion of cytokines triggers the clearance of the tissue from the debris and the activation of stem cells. Phagocytosis of apoptotic and necrotic cells induces an M1 to M2 macrophage transition (I). M2 polarized macrophages originate from resident macrophages (II) or can be recruited from circulating monocyte (III). This is a regenerative stage during which stem cells differentiate and the damage is resolved. In chronic diseases several rounds of damage and repair occur: both M1 and M2 polarized macrophages coexist in the tissue, recruited from monocytes. This persistent inflammation leads to fibrosis, fat deposition, and exhaustion of the stem cell pool.

Research in the past few years has highlighted a pivotal role of macrophages in tissue repair and remodelling. Macrophages are renowned for their plasticity and heterogeneity, which have been described not only* in vitro* but also in various physiological and pathological contests. Evidence indicated that macrophages are extremely versatile cells that can undergo phenotype changes according to specific environmental cues. In skeletal muscle, after acute injury, proinflammatory M1 macrophages firstly arrive to clear debris and are sequentially replaced by healing M2 macrophages that sustain tissue repair and regeneration. In chronic muscle injury, both M1 and M2 macrophages coexist but fail to promote tissue repair and homeostasis recovery (Figure 1). The efforts of the next years are likely to identify the molecular determinants of macrophage polarization in order to possibly develop effective targeted therapies for genetic defects of the tissue and muscle diseases associated with chronic inflammation.
